# DeepHeartCT: A fully automatic artificial intelligence hybrid framework based on convolutional neural network and multi-atlas segmentation for multi-structure cardiac computed tomography angiography image segmentation

**DOI:** 10.3389/frai.2022.1059007

**Published:** 2022-11-22

**Authors:** Vy Bui, Li-Yueh Hsu, Lin-Ching Chang, An-Yu Sun, Loc Tran, Sujata M. Shanbhag, Wunan Zhou, Nehal N. Mehta, Marcus Y. Chen

**Affiliations:** ^1^National Heart Lung and Blood Institute, National Institutes of Health, Bethesda, MD, United States; ^2^Radiology and Imaging Sciences, Clinical Center, National Institutes of Health, Bethesda, MD, United States; ^3^Department of Electrical Engineering and Computer Science, Catholic University of America, Washington, DC, United States

**Keywords:** cardiac computed tomography, heart segmentation, multi-atlas segmentation, convolutional neural network, deep learning

## Abstract

Cardiac computed tomography angiography (CTA) is an emerging imaging modality for assessing coronary artery as well as various cardiovascular structures. Recently, deep learning (DL) methods have been successfully applied to many applications of medical image analysis including cardiac CTA structure segmentation. However, DL requires a large amounts of data and high-quality labels for training which can be burdensome to obtain due to its labor-intensive nature. In this study, we aim to develop a fully automatic artificial intelligence (AI) system, named DeepHeartCT, for accurate and rapid cardiac CTA segmentation based on DL. The proposed system was trained using a large clinical dataset with computer-generated labels to segment various cardiovascular structures including left and right ventricles (LV, RV), left and right atria (LA, RA), and LV myocardium (LVM). This new system was trained directly using high-quality computer labels generated from our previously developed multi-atlas based AI system. In addition, a reverse ranking strategy was proposed to assess the segmentation quality in the absence of manual reference labels. This strategy allowed the new framework to assemble optimal computer-generated labels from a large dataset for effective training of a deep convolutional neural network (CNN). A large clinical cardiac CTA studies (*n* = 1,064) were used to train and validate our framework. The trained model was then tested on another independent dataset with manual labels (*n* = 60). The Dice score, Hausdorff distance and mean surface distance were used to quantify the segmentation accuracy. The proposed DeepHeartCT framework yields a high median Dice score of 0.90 [interquartile range (IQR), 0.90–0.91], a low median Hausdorff distance of 7 mm (IQR, 4–15 mm) and a low mean surface distance of 0.80 mm (IQR, 0.57–1.29 mm) across all segmented structures. An additional experiment was conducted to evaluate the proposed DL-based AI framework trained with a small vs. large dataset. The results show our framework also performed well when trained on a small optimal training dataset (*n* = 110) with a significantly reduced training time. These results demonstrated that the proposed DeepHeartCT framework provides accurate and rapid cardiac CTA segmentation that can be readily generalized for handling large-scale medical imaging applications.

## Introduction

Cardiac computed tomography angiography (CTA) is emerging as a non-invasively main-stream imaging modality for measuring the morphological changes of the heart and coronary arteries for diagnosing cardiovascular disease. CTA imaging opens new demands and opportunities on image analysis and clinical reporting. The quantitative assessment of different cardiovascular structures involves volumetric measurement of the ventricles, atria, left ventricular myocardium, and other great vessels from the CTA images. In practice, manual delineation by trained medical professionals remains the main approach to quantify these anatomical volume sizes on CTA images. This approach is tedious, subject to user variability, and very time consuming for domain experts to perform on large-scale clinical studies. A fully automatic segmentation which improves objectivity and reproducibility is highly desirable for the quantitative evaluation of different volumetric cardiac structures.

Accurate and reliable automatic whole heart segmentation in cardiac CTA remains challenging and is an active area of research (Zhuang et al., 2019; Habijan et al., 2020). A comprehensive review of existing automatic methods for cardiac CTA image segmentation was presented in (Bui et al., 2020a,b; Habijan et al., 2020) which includes several deep learning (DL) methods (Payer et al., 2017; Yang X. et al., 2017; Liu et al., 2019; Ye et al., 2019; Baskaran et al., 2020) and multi-atlas segmentation (MAS) methods (Kirişli et al., 2010; van Rikxoort et al., 2010; Zuluaga et al., 2013; Yang et al., 2014; Zhuang et al., 2015; Zhuang and Shen, 2016; Yang G. et al., 2017; Katouzian et al., 2018; Wang et al., 2018). Compared with MAS methods, DL based approaches have become more popular and demonstrated higher accuracy with a faster inference time. Zhao et al. proposed a DL method based on landmark registration and 3D fully convolutional network (Zhao et al., 2019). Several works used U-Net based architecture for whole heart segmentation in cardiac CTA (Payer et al., 2017; Liu et al., 2019; Ye et al., 2019; Baskaran et al., 2020). Vigneault et al. introduced Omega-Net consisting of a set of U-Net for heart segmentation in cardiac magnetic resonance (MR) images (Vigneault et al., 2018). Ye et al. presented a multi-depth fused with deeply supervised mechanism U-Net to segment seven cardiac structures in CTA images (Ye et al., 2019). Ding et al. proposed a CAB U-Net which added a category attention boosting module into U-Net to enhance the gradient flow in the network in cardiac MR and CTA applications (Ding et al., 2020). A semi-supervised Dual-Teacher and Dual-Teacher++ was introduced by Li et al. to use unlabeled and cross-modality data to segment cardiac CTA images (Li et al., 2020, 2021). A similar semi-supervised approach using few-shot learning was also presented by Wang et al. (2021).

Recently, deep learning (DL) based methods have shown great promise in medical image processing and analysis. They have achieved superior performance in various clinical applications with different imaging modalities (Shen et al., 2017). DL methods have the potential to provide faster segmentation and higher accuracy, compared to the conventional computer vision approaches, such as the deformable model-based algorithms and multi-atlas segmentation methods. However, DL requires a large amount of labeled data for training which is difficult to obtain in medical imaging field since the manual annotation requires significant time and efforts from domain experts. In practice, human observers evaluate the image quality and manual delineate over hundreds of slices in a CTA study to obtain ground truth segmentation. This process is extremely labor-intensive and can introduce bias and variability especially in large-scale cohort studies. Therefore, manually labeled data is costly, highly subjective and time consuming. On the other hand, while DL based methods may produce high segmentation efficiency and accuracy for in-domain data, they may cause extraneous regions and other non-physical artifacts when applied to cross-domain new data. Correcting such errors would require carefully designed post-processing steps and sometimes experts' manual efforts (Kong et al., 2021).

To overcome aforementioned shortcomings, we propose a fully automatic artificial intelligence (AI) hybrid framework, named DeepHeartCT, for multi-structure CTA segmentation which is trained by using high-quality computer-generated labels based on a label generator developed in our previous works (Bui et al., 2018, 2020a,b). The proposed framework combines several novel techniques includes multi-atlas segmentation, reverse ranking and convolutional neural network. The reverse ranking technique leverages a reverse classification scheme (Valindria et al., 2017) which measures the segmentation performance in the absence of reference labels. This allows the system to obtain a better subset of computer-generated labels to further improve the model performance. In addition, we will investigate the impact of the training data quality as well as quantity for the performance of the proposed framework based on a large cardiac CTA dataset.

## Materials and methods

### Clinical dataset

The dataset consists of 1,124 clinical cardiac CTA scans of patients with suspected cardiovascular diseases referred to National Heart, Lung, and Blood Institute (NHLBI). All CTA exams were performed under procedures and protocols approved by the Institutional Review Board of the National Institutes of Health. Written informed consent was obtained from all subjects prior to participating in the study. All CTA studies were performed on a 320-detector row scanner (Aquillion One Genesis, Canon Medical Systems, Japan) with 0.5 mm detector collimation, 275 msec gantry rotation time, 100–120 kVp tube voltage, 200–850 mA tube current according to patient's attenuation profile determined by the scout image. Contrast material dose was 50–70 mL administered at a flow rate of 5.0–5.5 mL/sec and adjusted for body habitus. Prospective ECG-triggered image acquisition was initiated by a target threshold of 350–400 HU in the descending aorta. For each dataset, images were reconstructed at a 75% phase window around diastole in the cardiac cycle with a matrix size of 512 × 512 and an average pixel size 0.36 × 0.36 (from 0.26 × 0.26 to 0.43 × 0.43) mm^2^. Each study contains 240–520 images with an average slice thickness of 0.33 (from 0.25 to 0.5) mm.

Among the 1,124 cases, 60 cases were manually labeled by two trained observers using a custom developed interactive image analysis software and reviewed by experienced cardiologists as the reference standard. There were 12 cardiovascular structures labeled in each study as described in our previous work (Bui et al., 2020a,b). In this paper, we will focus on five main cardiovascular structures which include left and right ventricular cavity (LV, RV), left and right atrial cavity (LA, RA), and left ventricular myocardium (LVM). The remaining 1,064 case were used to develop and train the proposed framework.

### DeepHeartCT: A hybrid multi-atlas, reverse ranking and CNN framework

Deep learning (DL) methods have been demonstrated great potential in whole heart segmentation, though several of them reported poor results in a blinded evaluation (Zhuang et al., 2019). The performance of DL-based methods could vary greatly across different network structures and training strategies. In addition, DL requires a large training dataset which is often difficult to obtain especially in medical field due to the availability of sufficiently large, curated, and representative training data that requires expert's labeling (e.g., annotations). On the other hand, conventional approaches, mainly based on multi-atlas segmentation (MAS), showed a reliable and stable performance while only required a smaller training dataset, though the accuracy and computational efficiency is often lower than DL approaches. However, as demonstrated in our previous works (Bui et al., 2020a,b), the combined multi-atlas and corrective segmentation (CMACS) framework based on the multi-atlas approach does not suffer from reduced accuracy as well as computational deficiency like in previous MAS-based methods.

**Figure 1:**
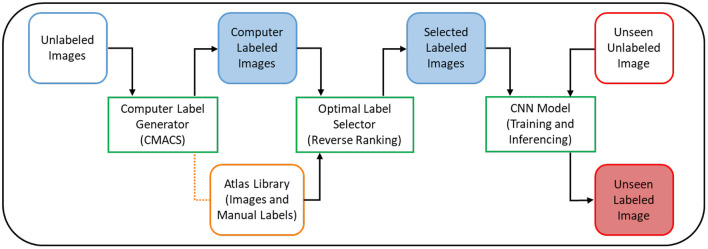
Flow diagram of the proposed DeepHeartCT framework: A hybrid CMACS and CNN based framework for fully automatic cardiac CTA image labeling, quality assurance, fast and accurate segmentation.

In this work, we proposed to use our previous CMACS framework as a computer label generator to create labeled dataset for DL training, thus remove the need for domain experts to manual label and contour the training dataset. In addition, we presented a reverse ranking (RR) technique to evaluate the quality of these computer-generated labels in the absence of manual labels. This RR approach is designed to grade the quality of computer generated labels, so to remove less accurate labels and retain the higher quality ones for DL training. In our implementation, we utilized a U-net based deep learning architecture, trained it on high-quality computer generated labels, and validated the model on expert manual labels. Figure 1 shows the flow diagrams of the proposed DeepHeartCT framework which is a hybrid system combining the CMACS and convolutional neural network (CNN) for fully automatic cardiac CTA image segmentation. Each green block in Figure 1 will be further elaborated in the following subsections.

#### Computer label generator

The CLG of the DeepHeartCT framework was part of our CMACS framework (Bui et al., 2018, 2020a,b). The CMACS framework consists of three core processing blocks: (1) bounding box detection, (2) multi-atlas segmentation, and (3) corrective segmentation, to perform simultaneous multi-structure heart and peripheral tissue segmentation.

First, the bounding box detection is used to identify the confined region that contains the whole heart. A series of image processing steps based on supervoxel and region segmentation are used to identify the six faces of a 3D bounding box containing the whole heart in a CTA image volume. Second, the multi-atlas segmentation begins with a fast strategy to select an optimal subset of atlases from an atlas library. This is performed by matching structural similarities (Achanta et al., 2012) between a given target image and all images in the atlas library to select an optimal set of nine atlas images. A pairwise atlas-to-target deformable image registration is then performed on each selected atlas to obtain a non-linear transformation to warp the associated atlas label into the target image space (Heinrich et al., 2013). After the multi-atlas registration, a label fusion scheme is used to merge the warped labels into a single consensus target label. Finally, the corrective segmentation is designed to refine the cardiovascular labels obtained from the previous processing steps and to separate the intrathoracic tissue structures surrounding the heart. It begins with automated image processing steps to extract representative seed voxels from non-cardiac structures that include lung, chest wall, livers, spine, and descending aorta. Together with the previously obtained cardiac structures, a random walk algorithm (Grady, 2006) is then performed on each seed region in a multiple-pass fashion to improve the segmentation result for each structure. The final segmentation is obtained by additional post-processing steps using morphology and connected region analysis for further refinement. The quality of these computer-generated labels is then assessed using an optimal label selector which is described in the next section.

#### Optimal label selector based on reverse ranking

**Figure 2:**
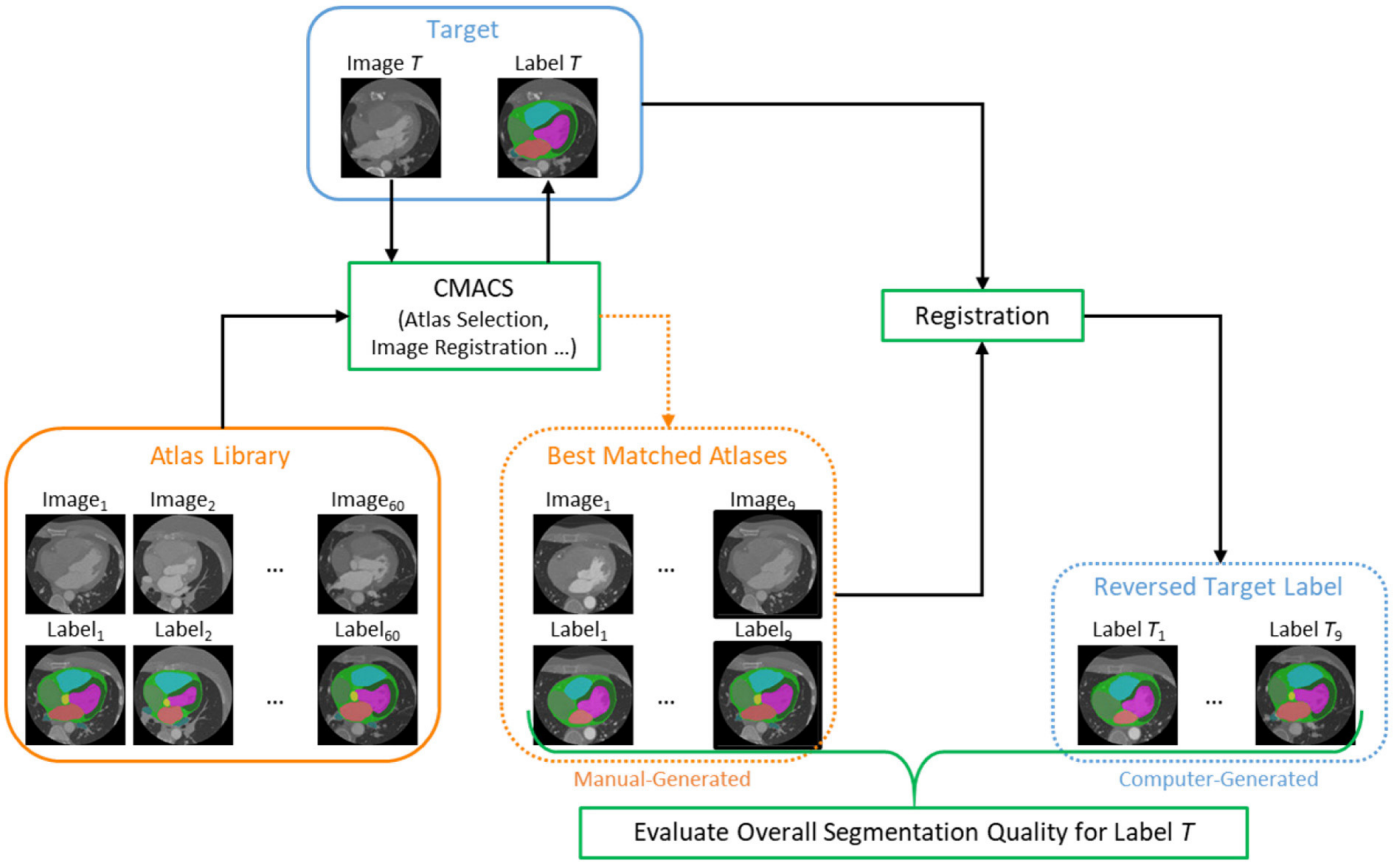
Flow diagram of the proposed Optimal Label Selector (OLS) using reverse ranking to rank the segmentation accuracies for all cardiac CTA clinical studies (*n* = 1,064).

After the unlabeled images (*n* = 1,064) were processed through the CLG, the next step is to evaluate the segmentation quality of all images using a reverse ranking (RR) approach to score and obtain a subset of images with higher segmentation quality. This approach was inspired from the reverse classification accuracy introduced by Valindria et al. (2017) that is useful to assess the segmentation quality in the absence of manual reference labels. The flow diagram of the proposed Optimal Label Selector (OLS) is shown in Figure 2. For each unlabeled (or target) image, CMACS is used to obtain the computer-generated label. The quality of computer label is then measured by registering the target image acts as moving image with nine selected atlases which act as fixed image to obtain nine reversed target labels. These nine atlases were selected by matching structural similarities technique described in (Bui et al., 2020a) from a library of 60 manually generated atlases which have the ground truth labels performed by human experts. We can then evaluate the segmentation quality of the target image by calculating the average Dice values of these nine reversed target labels and their corresponding manual labels. Such average Dice value is defined as RR score in the subsequent sections.

#### CNN architecture: U-net based

The proposed DeepHeartCT framework utilized the well-known U-net model to generate the labels (Ronneberger et al., 2015; Çiçek et al., 2016). In our implementation, the model can be customized to segment up to 12 structures simultaneously. The bounding box detection was used to crop the raw image volume before extracting the patches as localizing the heart would reduce the number of false positive voxels. By processing the image within the bounding box only, the total number of voxels was reduced for segmentation, thus further reducing the training and prediction time. Generally, a bigger patch yields a better result in DL. However, due to the GPU hardware limitation, the input patch size was empirically set to 64 × 100 × 100 with a stride 32 × 64 × 64 between patches. Each layer contains a convolutional 3D layer and ReLU followed by a group norm. Dice coefficient was used as the loss function, as is commonly used for binary semantic segmentation. Since more than two classes are present in the ground truth, the Dice loss per channel was computed and then averaged to obtain the final value. Sigmoid was used after the final layer to normalize the prediction score to probability score (0, 1). We used sigmoid activation function because the target segmentation is a binary task for each label, not a multiclass segmentation task. There are five binary segmentations, we prioritize the labels in the following order: LV, LA, RV, RA, LVM, and then combine them for the final results. In this DeepHeartCT framework, multi-step learning rate (Paszke et al., 2017) was used as the learning rate scheduler with milestones set to (van Rikxoort et al., 2010; Heinrich et al., 2013; Zuluaga et al., 2013; Zhuang et al., 2015; Payer et al., 2017; Shen et al., 2017; Yang G. et al., 2017; Vigneault et al., 2018; Wang et al., 2018; Liu et al., 2019; Bui et al., 2020b; Habijan et al., 2020) and gamma parameter set to 0.1. Adam stochastic algorithm (Kingma and Ba, 2015) was used as optimizer with an initial learning rate starts at 0.0001 and weight decay at 0.0001. Batch size, feature maps scale factor, and number of group norm were empirically set to 1, 64 and 16, respectively. The number of epochs was set to 100.

### Evaluation methods

To evaluate the segmentation performances of DeepHeartCT, we investigated four different settings of the training dataset:

(1) All (All) dataset: CNN trained on all computer labels generated by CMACS without OLS to exclude any label (*n* = 1,064).
(2) Strong Label (SL) dataset: CNN trained on the computer labels generated by CMACS with OLS to select the best 10% of labels (*n* = 110).
(3) Weak Label (WL) dataset: CNN trained on the computer labels generated by CMACS with OLS to select the worst 10% of labels (*n* = 110).
(4) Synthetic Weak Label (sWL) dataset: a synthetic weak label dataset was artificially created from the SL dataset by performing 5 mm erosion on the four cardiac chambers, and then expanding the LVM to cover the entire LV cavity to create the synthetic labels (*n* = 110).

In our evaluation, we intentionally degraded the labels quality and created the sWL dataset since the WL dataset's quality, even though selected from the lowest reverse ranking scores, is still very reasonable. This sWL dataset allows us to evaluate the effectiveness of the proposed OLS and compare their performance against good labels for CNN training. A qualitative example of the Strong Label and Synthetic Weak Label is shown in Supplementary Figure 1. Four different models were built using these four training datasets with the same CNN hyperparameters under the DeepHeartCT framework for comparisons.

The model performance was evaluated using five-fold cross validation. As shown in Table 1, The All dataset (*n* = 1,064) was divided into 851 cases for training and 213 cases for validation, while SL, WL, and sWL (*n* = 110) dataset was divided into 88 cases for training and 22 cases for validation. After the training, all four models were tested using the independent manual label dataset (*n* = 60) for performance evaluation. To further compare the performance of the four models, a public domain dataset provided by the Multi-Modality Whole Heart Segmentation (MMWHS) challenge (Zhuang et al., 2019) which included 20 cardiac CTA images with manual segmentation from an independent institution was evaluated. This dataset was acquired from different CT scanners and with different imaging parameters to test the generalizability of our models.

**Table 1:** Training, validation, and testing sample size for the proposed DeepHeartCT framework based on All, strong label (SL), weak label (WL), and synthetic weak label (sWL) dataset.

**Dataset**	**Training samples (cases)**	**Validation samples (cases)**	**Testing samples (cases)**
All	851	213	60
SL, WL, sWL	88	22	60

The proposed framework was implemented in Python and Interactive Data Language (Harris Geospatial Solutions). The registration method was developed in C++ by Heinrich et al. (2013) and compiled to dynamic link library under Microsoft Visual Studio in our framework. All studies were processed with the same parameter settings on a computer with an Intel Core i9-10980XE 3.00GHz CPU, NVIDIA GeForce RTX 3090, and 128GB RAM.

For the performance evaluation of the automated segmentation, three quantitative metrics that measure the differences between the automatic results vs. the corresponding manual labels are computed which include Dice coefficient (Dice), Hausdorff distance (HD) and mean surface distance (MSD). Summary statistics of the results are expressed as the median and 95% confidence interval for non-normally distributed data and as mean and standard deviation for normally distributed data. A *p* < 0.05 indicates a statistically significant difference.

## Results

**Figure 3:**
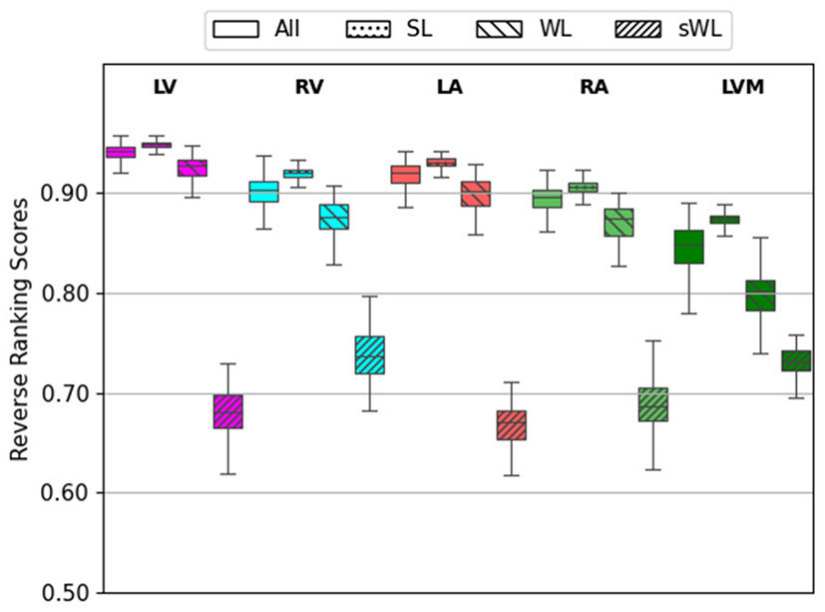
Average reverse ranking (RR) scores computed using the labels from Computer Label Generator (CLG). RR scores are plotted for each structure with the four datasets in the order of All, strong label (SL), weak label (WL), and synthetic weak label (sWL).

An important new feature in the proposed DeepHeartCT framework is the new OLS block to select an optimal subset of computer-generated labels for training, as described in the section Optimal Label Selector based on Reverse Ranking. Figure 3 shows the average reserve ranking (RR) scores for the five segmented structures (LV, RV, LA, RA, and LVM) from the four datasets, i.e., All (*n* = 1,064), SL (*n* = 110), WL (*n* = 110), sWL (*n* = 110).

As the overall segmentation quality assessed using the reverse ranking scheme in OLS for the entire (All) dataset was mostly excellent to good, with average RR scores ranged from 0.84 to 0.94 in five segmented structure. For this reason, we created the synthetic weak label (sWL) dataset to evaluate the effectiveness of our RR scheme to grade labels with lower quality. As shown in Figure 3, it is clear that DeepHeartCT-SL has the highest RR score with a median value of 0.92 (IQR, 0.91–0.92) within all five structures. DeepHeartCT-All and DeepHeartCT-WL have slightly lower scores with the median RR score of 0.90 (IQR, 0.87–0.92) and 0.88 (IQR, 0.77–0.88), respectively. This result is expected because the SL dataset was selected from the top 10% of the entire dataset based on the RR score. Finally, the sWL dataset has the lowest score with the median RR score of 0.70 (IQR, 0.68–0.72) which is also expected because we manipulated the segmentation quality to obtain these weak labels. Figure 3 demonstrates that the proposed OLS with reversed ranking technique is effective for assessing the segmentation quality in the absence of manual labels.

**Figure 4:**
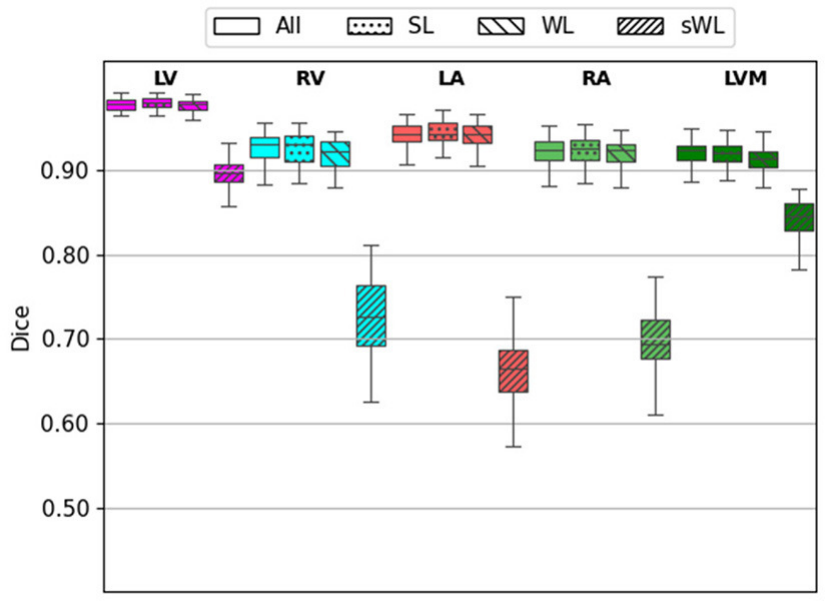
Dice score calculated from the testing of 60 independent cardiac CTA studies comparing the four trained models based on the All, strong label (SL), weak label (WL), and synthetic weak label (sWL) dataset. Average Dice score is reported for each structure including left and right ventricles (LV, RV), left and right atria (LA, RA), and LV myocardium (LVM).

Next, we evaluated the segmentation accuracy of four trained models using the 60 independent CTA studies that were manually annotated by the domain experts. Figure 4 shows the Dice score for each structure comparing the four trained models. DeepHeartCT-All yielded a median Dice of 0.98 (IQR, 0.97–0.99) for LV, 0.94 (IQR, 0.90–0.96) for RV, 0.94 (IQR, 0.92–0.96) for LA, 0.93 (IQR, 0.88–0.95) for RA, and 0.93 (IQR, 0.89–0.94) for LVM. DeepHeartCT-SL yielded a median Dice of 0.98 (IQR, 0.97–0.99) for LV, 0.94 (IQR, 0.90–0.96) for RV, 0.95 (IQR, 0.92–0.97) for LA, 0.93 (IQR, 0.87–0.95) for RA, and 0.92 (IQR, 0.88–0.94) for LVM. DeepHeartCT-WL yielded a median Dice of 0.98 (IQR, 0.96–0.99) for LV, 0.92 (IQR, 0.89–0.94) for RV, 0.94 (IQR, 0.91–0.96) for LA, 0.92 (IQR, 0.88–0.94) for RA, and 0.91 (IQR, 0.87–0.93) for LVM. DeepHeartCT-sWL yielded a median Dice of 0.67 (IQR, 0.63–0.71) for LV, 0.71 (IQR, 0.65–0.78) for RV, 0.68 (IQR, 0.59–0.73) for LA, 0.70 (IQR, 0.61-0.74) for RA, and 0.73 (IQR, 0.66–0.78) for LVM. Furthermore, Supplementary Figure 2 shows the Dice score comparison based on the MMWHS dataset.

**Figure 5:**
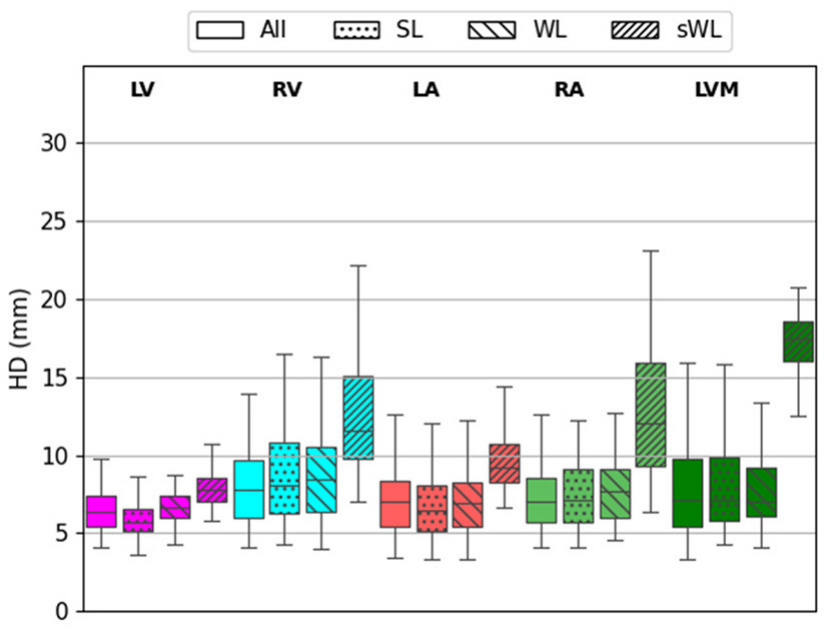
Hausdorff distance (HD) calculated from the testing of 60 independent cardiac CTA studies comparing the four trained models based on the All, strong label (SL), weak label (WL), and synthetic weak label (sWL) dataset. Average HD is reported in mm for each structure including left and right ventricles (LV, RV), left and right atria (LA, RA), and LV myocardium (LVM).

Figure 5 shows the HD error measurements for the five segmented structures using four different models. For the DeepHeartCT-All model, the median HD was 6 mm (IQR, 4–8 mm) for LV, 7 mm (IQR, 4–16 mm) for RV, 6 mm (IQR, 4–11 mm) for LA, 7 mm (IQR, 4–11 mm) for RA, and 8 mm (IQR, 4–17 mm) for LVM. For the DeepHeartCT-SL model, the median HD was 5 mm (IQR, 4–8 mm) for LV, 8 mm (IQR, 4–19 mm) for RV, 6 mm (IQR, 4–14 mm) for LA, 7 mm (IQR, 4–16 mm) for RA, and 9 mm (IQR, 5–18 mm) for LVM. DeepHeartCT-WL yielded a median HD of 7 mm (IQR, 5–9 mm) for LV, 8 mm (IQR, 5–16 mm) for RV, 7 mm (IQR, 4–11 mm) for LA, 8 mm (IQR, 5–13 mm) for RA, and 7 mm (IQR, 5–12 mm) for LVM. For the DeepHeartCT-sWL model, the median HD was 11 mm (IQR, 9–17 mm) for LV, 10 mm (IQR, 8–21 mm) for RV, 9 mm (IQR, 6–16 mm) for LA, 12 mm (IQR, 8–21 mm) for RA, and 18 mm (IQR, 16–20 mm) for LVM. Furthermore, Supplementary Figure 3 shows the HD error comparison based on the MMWHS dataset.

**Figure 6:**
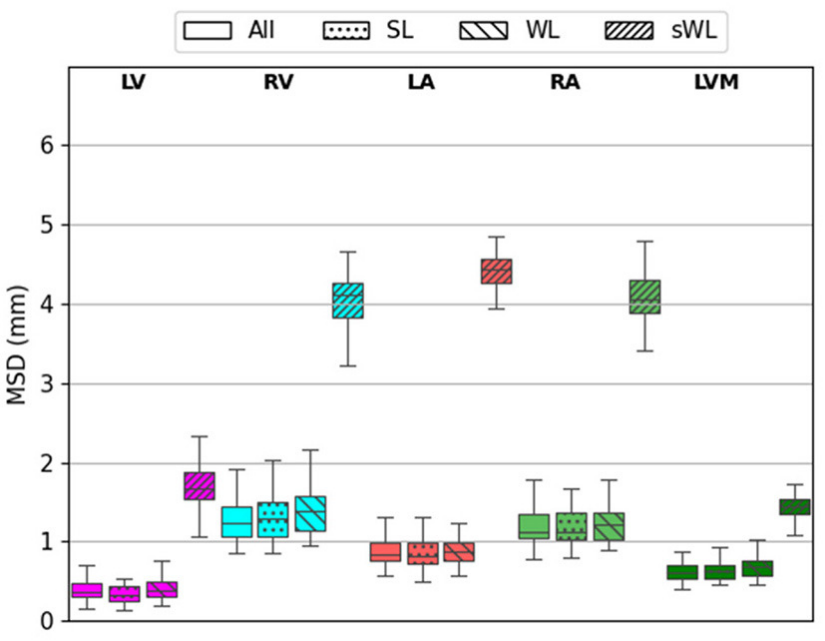
Mean surface distance (MSD) calculated from the testing of 60 independent cardiac CTA studies comparing four trained models based on the All, strong label (SL), weak label (WL), and synthetic weak label (sWL) dataset. Average MSD is reported in mm for each structure including left and right ventricles (LV, RV), left and right atria (LA, RA), and LV myocardium (LVM).

Similarly results were found on the MSD error measurements for the five segmented structures. As shown in Figure 6, DeepHeartCT-All yielded a median MSD of 0.34 mm (IQR, 0.21–0.59 mm) for LV, 1.14 mm (IQR, 0.88–1.68 mm) for RV, 0.89 mm (IQR, 0.61–1.21 mm) for LA, 1.14 mm (IQR, 0.89–1.74 mm) for RA, and 0.61 mm (IQR, 0.45–0.78 mm) for LVM. DeepHeartCT-SL had a median MSD of 0.31 mm (IQR, 0.16–0.57 mm) for LV, 1.14 mm (IQR, 0.83–1.71 mm) for RV, 0.81 mm (IQR, 0.55–1.22 mm) for LA, 1.14 mm (IQR, 0.86–2.00 mm) for RA, and 0.59 mm (IQR, 0.46–0.93 mm) for LVM. DeepHeartCT-WL yielded a median MSD of 0.39 mm (IQR, 0.24–0.61 mm) for LV, 1.39 mm (IQR, 1.04–1.89 mm) for RV, 0.87 mm (IQR, 0.63–1.21 mm) for LA, 1.22 mm (IQR, 0.94–1.73 mm) for RA, and 0.68 mm (IQR, 0.47–1.01 mm) for LVM. DeepHeartCT-sWL generated a median MSD of 5.1 mm (IQR, 4.6–5.5 mm) for LV, 4.3 mm (IQR, 3.6–5.1 mm) for RV, 4.2 mm (IQR, 3.6–4.5 mm) for LA, 4.1 mm (IQR, 3.3–4.6 mm) for RA, and 2.5 mm (IQR, 2.4–2.8 mm) for LVM. Furthermore, Supplementary Figure 4 shows the MSD error comparison based on the MMWHS dataset.

Overall, these quantitative results show that DeepHeartCT-All and DeepHeartCT-SL had a compatible performance for segmenting the five main cardiac structures in CTA image. However, DeepHeartCT-SL reduces the total CNN training time by using a smaller but better samples. Finally, the segmentation performance from both DeepHeartCT-All and DeepHeartCT-SL models is significantly better than the DeepHeartCT-sWL model.

A comparison of the training and inference time between DeepHeartCT-All and DeepHeartCT-SL is summarized in Table 2 with the same hyper-parameters settings. While the inference time was similar between DeepHeartCT-All and DeepHeartCT-SL models, the required training time was significantly reduced from 90 min in DeepHeartCT-All to only 50 min in DeepHeartCT-SL.

**Table 2:** Computational time comparison for the proposed DeepHeartCT frameworks using All and strong label (SL) dataset.

**Dataset**	**Training samples (cases)**	**GPU training time (mins)**	**GPU inference time (secs)**	**CPU inference time (secs)**
All	1,064	90	20.6 ± 4.80	32.0 ± 7.79
SL	110	50	21.9 ± 4.81	34.2 ± 7.04

## Discussions

In this study, we have presented a new AI framework for rapid and automated multi-structures segmentation from cardiac CTA scans for image labeling, quality assurance, and accurate segmentation. The proposed DeepHeartCT framework significantly reduces the effort for manually annotating a large dataset for CNN training which was a bottleneck in many medical image analysis applications because obtaining such gold-standard labels is often very time-consuming, especially in large-scale clinical studies or in the studies where multiple annotated labels are needed for different anatomic structures such as cardiac CTA images.

We have addressed three main challenges in CNN-based medical image segmentation using DL for large clinical studies. First, training labels for CNN require domain expertise for manual contouring or masking of the images. Second, CNN requires not only a large amount of labeled data for training but also high quality labels. Finally, a long training time for CNN is often required with a larger sample size. We tackled the first challenge by exploiting large quantity of computer-generated labels through the CMACS processing. Next, we employed a novel reverse ranking (RR) approach to evaluate the quality of these computer labels in the absence of ground truth labels. Finally, we presented an optimal label selector based on the proposed RR score to attain only high-quality computer labels to speed up CNN training while maintain a similar or improved accuracy. By selecting good labels for training, we can lessen the influences of low-quality computer labels on training the CNN.

As shown in Figures 4–6, the segmentation results among DeepHeartCT-All, DeepHeartCT-SL, DeepHeartCT-WL, and DeepHeartCT-sWL were compared using three different quantitative metrics. A comparable Dice score of 0.94 was obtained in most of the structures when selecting only the high-quality dataset (DeepHeartCT-SL) from a large computer labeled dataset (DeepHeartCT-All) for CNN training. While DeepHeartCT-SL shows no improvement in term of HD compared to DeepHeartCT-All, it shows an average of 3.2% improvement in the MSD measurements. To compare DeepHeartCT-SL and DeepHeartCT-sWL, an average of 34.9% improvement in the Dice score was observed. Furthermore, there was an average of 41.6% and 80.1% improvement in HD and MSD error measurements, respectively (see Figures 5, 6). A similar trend in performance comparison was observed in Supplementary Figures 2–4 when testing the four models on the MMWHS dataset from another institution. These results may be explained by the fact that the CNN performs better with more accurate labels as it does not need to account for the poor segmentation labels.

In general, a larger amount of training samples is desired in deep learning but a longer training time is also expected. It was also known that more training data can lead to lower estimation variance and hence a better model performance. More labeled data also increases the probability of useful information in CNN training. However, more labeled data does not always helpful if the quality of the labels is suboptimal or noisy. With the proposed OLS approach and RR technique, we are able to reduce the CNN training time while maintain a similar or better segmentation accuracy by using a smaller but better quality training dataset.

For the training time comparison (see Table 2), the DeepHeartCT-SL framework took about 50 min which is about 45% faster than the DeepHeartCT-All. For the inference time, DeepHeartCT is comparable with other DL-based methods as presented in the Multi-Modality Whole Heart Segmentation Challenge (Zhuang et al., 2019). The inference time of DeepHeartCT is also considerably faster than our previous CMACS framework which took about 2 min (Bui et al., 2020b), and is significantly faster than other multi-atlas based methods which have been reported to take around 21 min (Zhuang et al., 2019).

There were existing methods which aim at identifying, fixing or discarding training samples that are likely to have incorrect labels (Karimi et al., 2020). Vo et al. proposed supervised and unsupervised image ranking methods for identifying correctly labeled images in a large quantity of images with noisy labels (Vo et al., 2015). Their proposed methods were based on matching each image with a noisy label to a set of representative images with clean labels. Xue et al. also proposed a self-supervised method to regularize the networks to utilize noisy samples in medical image classification task (Xue et al., 2022). To our best knowledge, this work is the first study to leverage the reverse ranking strategy to evaluate the segmentation quality without the ground truth labels. This approach enables us to exploit a large number of computer labels without human manual efforts and effectively mitigate the impact of weak labels in training neural networks.

There are some limitations in our study. All studies were retrospectively collected from a single center and from a single vendor, and all images includes only a single-phase (i.e., 75% time point) of the cardiac cycle. Our work did not include patients with congenital heart defects such as single ventricle, atrial and ventricle septal defects, or other abnormal cardiac structures, or under cardiovascular surgeries. We only selected a subset of high quality labels based on the top 10% RR score rather than a more comprehensive selection of different subset sizes for performance and speed evaluations.

In summary, the proposed DeepHeartCT framework includes a CLG block to provide computer generated labels from a large clinical dataset with over a thousand cardiac CTA scans. This framework also includes a novel OLS block to automatically select high-quality labels for CNN training, and improve the training speed and model performance for multi-structure cardiac CTA image segmentation. Our quantitative comparisons based on an independent dataset showed a strong agreement between DeepHeartCT automatic and expert manual segmentation for all five cardiac structures assessed. These results demonstrated that the proposed fully automatic AI system provides high-quality and rapid cardiac CTA segmentation that can be readily generalized for processing large-scale datasets for further clinical applications.

## Data availability statement

The dataset presented in this article is not readily available due to patient confidentiality. Requests to access the dataset should be directed to the corresponding author at lyhsu@nih.gov.

## Ethics statement

The studies involving human participants were reviewed and approved by the Institutional Review Board of the National Institutes of Health. The patients/participants provided their written informed consent to participate in the studies.

## Author contributions

VB and L-YH conceived the experiments. VB, L-YH, and L-CC developed the computer algorithms and software and drafted the manuscript. VB, L-YH, A-YS, LT, and L-CC analyzed the data. SS and MC supervised the image acquisition. SS, WZ, NM, and MC developed clinical research protocols. All authors have made substantive intellectual contribution to the work and approved the manuscript.

## Funding

This work was supported by the Intramural Research Program of the National Heart Lung and Blood Institute and Clinical Center of the National Institutes of Health.

## Conflict of interest

The authors declare that the research was conducted in the absence of any commercial or financial relationships that could be construed as a potential conflict of interest.

## Publisher's note

All claims expressed in this article are solely those of the authors and do not necessarily represent those of their affiliated organizations, or those of the publisher, the editors and the reviewers. Any product that may be evaluated in this article, or claim that may be made by its manufacturer, is not guaranteed or endorsed by the publisher.

## Abbreviations

AI: artificial intelligence
CLG: computer label generator
CNN: convolutional neural network
CTA: computed tomography angiography
DL: deep learning
GPU: graphics processing unit
HD: Hausdorff distance
IQR: interquartile range
LA: left atrium
LV: left ventricle
LVM: LV myocardium
MR: magnetic resonance
MAS: multi-atlas segmentation
MMWHS: Multi-Modality Whole Heart Segmentation
MSD: mean surface distance
NHLBI: national heart lung and blood institute
OLS: optimal label selector
RA: right atrium
RV: right ventricle
RR: reverse ranking
SGD: stochastic gradient descent
SL: strong label
sWL: synthetic weak label
WL: weak label.
