# Supplementary material for: DeepHeartCT: A fully automatic artificial intelligence hybrid framework based on convolutional neural network and multi-atlas segmentation for multi-structure cardiac computed tomography angiography image segmentation
Source: Front Artif Intell. 2022 Nov 22;5:1059007. doi: 10.3389/frai.2022.1059007 (PMC9723331; doi:10.3389/frai.2022.1059007)
Supplement: Supplementary file 1 [file Data_Sheet_1.docx]

# **Supplemental Material**


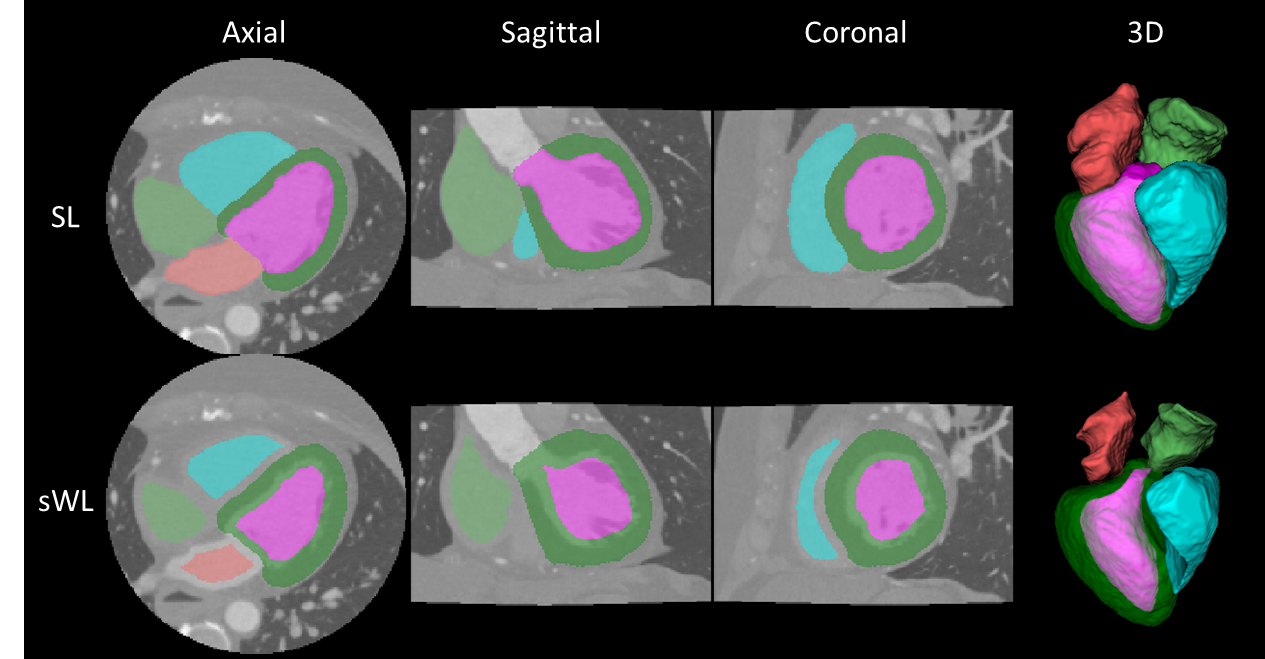


**Supplemental Figure 1** Qualitative example of the strong label (SL) and the synthetic weak label (sWL). Color labels: **LV**, **RV**, **LA**, **RA, LVM**.


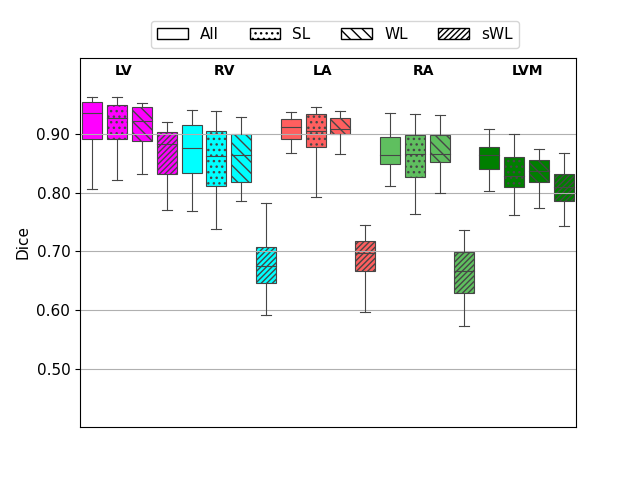


**Supplemental Figure 2** Dice score from the testing of MMWHS dataset comparing the four trained models based on the All, strong label (SL), weak label (WL), and synthetic weak label (sWL) dataset. Average Dice score is reported for each structure including left and right ventricles (LV, RV), left and right atria (LA, RA), and LV myocardium (LVM).


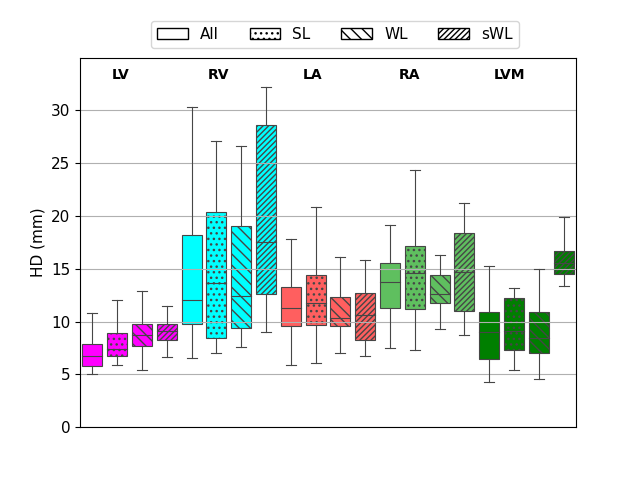


**Supplemental Figure 3** Hausdorff distance (HD) from the testing of the MMWHS datatset comparing the four trained models based on the All, strong label (SL), weak label (WL), and synthetic weak label (sWL) dataset. Average HD is reported in mm for each structure including left and right ventricles (LV, RV), left and right atria (LA, RA), and LV myocardium (LVM).


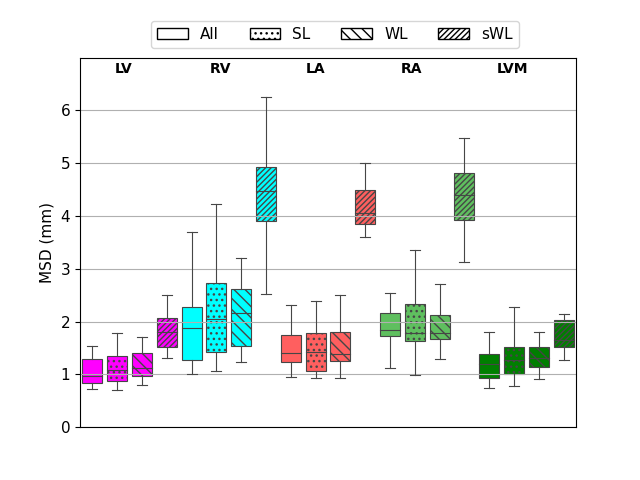


**Supplemental Figure 4** Mean surface distance (MSD) from the testing of MMWHS dataset comparing four trained models based on the All, strong label (SL), weak label (WL), and synthetic weak label (sWL) dataset. Average MSD is reported in mm for each structure including left and right ventricles (LV, RV), left and right atria (LA, RA), and LV myocardium (LVM).
